# The role of honey in the ecology of the hive: Nutrition, detoxification, longevity, and protection against hive pathogens

**DOI:** 10.3389/fnut.2022.954170

**Published:** 2022-07-25

**Authors:** Kenya E. Fernandes, Elizabeth A. Frost, Emily J. Remnant, Kathleen R. Schell, Nural N. Cokcetin, Dee A. Carter

**Affiliations:** ^1^School of Life and Environmental Sciences, University of Sydney, Sydney, NSW, Australia; ^2^Animal Genetics & Breeding Unit (ABGU), A Joint Venture of NSW Department of Primary Industries and University of New England, Armidale, NSW, Australia; ^3^NSW Department of Primary Industries, Paterson, NSW, Australia; ^4^Australian Institute for Microbiology and Infection, University of Technology, Sydney, NSW, Australia; ^5^Sydney Institute for Infectious Diseases, University of Sydney, Sydney, NSW, Australia

**Keywords:** honey, honey bee, honey bee ecology, medicinal honey, hive pathogens

## Abstract

Honey is the source of energy for the European honey bee, *Apis mellifera*. Beyond simple nutrition and a hedge against the seasonal, geographic, and chemical unpredictability of nectar, honey has properties that protect the hive against various stresses. Enzyme-mediated detoxification during honey ripening neutralizes potentially toxic phytochemicals, and bees that consume honey have enhanced tolerance to other ingested toxins. Catalase and antioxidant phenolics protect honey bees from oxidative damage caused by reactive oxygen species, promoting their longevity. Phytochemical components of honey and microRNAs have the potential to influence developmental pathways, with diet playing a large role in honey bee caste determination. Components of honey mediate stress response and promote cold tolerance during overwintering. Honey has a suite of antimicrobial mechanisms including osmotic pressure, low water activity, low pH, hydrogen peroxide, and plant-, honey bee-, and microbiota-derived compounds such as phytochemicals and antimicrobial peptides. Certain types of honey, particularly polyfloral honeys, have been shown to inhibit important honey bee pathogens including the bacteria responsible for American and European Foulbrood, the microsporidian *Nosema ceranae*, and the fungi responsible for Stonebrood. Understanding the diverse functional properties of honey has far-ranging implications for honey bee and hive health and management by beekeepers.

## Introduction

The European honey bee, *Apis mellifera*, possesses an extraordinary ability to process and store nectar in the form of honey. In the context of human health, honey is a highly valued natural product that has been used as a food and to treat various ailments since ancient times (1). Over recent decades, the specific nutritional and therapeutic effects of honey have been the focus of much research interest. Various constituents of honey have been shown to have a suite of therapeutically beneficial properties including antimicrobial, antioxidant, anti-inflammatory, and *in vivo* anticancer effects (2). Although there has been less research into the ecological role of honey, it has clearly evolved these functional properties to protect itself and the hive from microbial attack and spoilage, and to enhance honey bee health. In addition to producing honey, honey bees play a crucial role in agriculture, providing pollination services for a wide variety of crops globally (3).

Major challenges to global honey bee populations over recent decades including threats ranging from pesticides and pathogens to global warming and habitat transformation (4), and the imperative to reduce the use of chemical and antimicrobial agents to treat hive diseases, makes understanding the diverse functions of honey in the hive more important than ever. With our understanding of the complex actions of components of honey continually growing [reviewed by (5)], and the contribution of various colony products to honey bee health being increasingly recognized [reviewed by (6)], the fact that honey is a multi-functional food for honey bees, impacting far more than nutrition alone, has become clear [reviewed by (7)]. Here, we summarize research on the features and components of honey with a focus on its functional and ecological significance to honey bees and the hive (Table 1), including the various antimicrobial mechanisms and studies that have assayed honey against hive pathogens.

**Table 1:** Ubiquitous honey components and their ecological benefit to the hive.

**Honey Component**	**Origin**	**Function**	**Ecological benefit**
**Primarily bee-derived**			
β-glucosidase	Bee (hypopharyngeal gland secretions)	Enzyme that breaks down ingested glucosic toxins.	Protects bees by neutralizing potentially toxic phytochemicals present in nectar.
Diastase	Bee (hypopharyngeal gland secretions) and plant (nectar)	Enzyme that breaks down starch and dextrins into smaller carbohydrates.	Theorized to take part in pollen digestion.
Glucose-oxidase	Bee (hypopharyngeal gland secretions) and plant (nectar)	Enzyme that breaks down glucose in the presence of water, yielding gluconic acid and hydrogen peroxide.	Protects honey from microbial decomposition until a high enough sugar concentration is achieved to inhibit microbes via osmotic pressure.
Invertase	Bee (hypopharyngeal gland secretions) and plant (nectar)	Enzyme that breaks down sucrose in nectar into fructose and glucose in the final honey.	Results in honey being a highly energetic food occupying minimal space within the comb.
Bee defensin-1	Bee (hypopharyngeal gland secretions)	Antimicrobial peptide that has additive activity with hydrogen peroxide, sugars, and low pH.	Prevents microbial growth, protecting honey from spoilage and increasing storage life.
Major royal jelly protein-1	Bee (hypopharyngeal gland secretions)	Protein that yields antimicrobial peptides upon cleavage.	Prevents microbial growth, protecting honey from spoilage and increasing storage life.
Organic acids	Primarily produced by bees during the ripening process from nectar sugars, some directly from plant (nectar)	Approx. 30 organic acids with various functions.	Increase the acidity of honey contributing to making it difficult for microbes to grow.
**Primarily plant-derived**			
Amino acids	Bee (hypopharyngeal gland secretions) and plant (nectar and pollen)	Approx. 26 amino acids with various functions.	Contribute to antioxidant properties of honey.
Catalase	Plant (nectar and pollen)	Enzyme that breaks down hydrogen peroxide to water and oxygen.	Protects bees from oxidative damage caused by elevated hydrogen peroxide levels.
Phenolic compounds	Plant (nectar and pollen)	Various functions including inhibiting ROS.	Protects bees from oxidative damage.
Phytochemicals	Plant (nectar and pollen)	Numerous diverse chemicals with various functions.	Influence developmental pathways and contribute to caste determination through bee diet.

## The advantages of processing and storing nectar as honey

### Stable and efficient long-term storage

Nectar is the main source of energy for the hive in the form of carbohydrates and is primarily composed of sugars with varying levels of moisture, mineral content, enzymes, and phytochemicals depending on the floral source (5). Once transported back to the hive by foragers, nectar is deposited into the wax cells of the honeycomb where the physicochemical transformation into honey, known as ripening, takes place (6). During this process, worker bees continue to manipulate the developing honey by secreting various enzymes from their hypopharyngeal glands into the nectar, and these can metabolize its components. The most important is invertase, which converts sucrose into fructose and glucose creating a stable, high density, and highly energetic food source that occupies a minimum amount of space within the wax cells of the hive (7). Other enzymes include protease, which breaks down proteins and polypeptides to yield smaller peptides that may influence the quality and nutritional value of honey (8), and diastase, which breaks down starch and dextrins into smaller carbohydrates and is thought to play a role in the digestion of pollen, the main source of protein for honey bees (9).

While this biochemical processing is taking place, the moisture content of the nectar is also being minimized through active and passive evaporation, making it resilient to microbial spoilage and prolonging its storage life. Active evaporation behavior by worker bees includes increasing the surface area of the nectar by sucking up, regurgitating, and holding it between their mandibles (6), and wing-fanning to increase circulation throughout the hive (10), which is done until the water content reaches ~50–60%. It is then placed into wax cells and relocated periodically, evaporating passively until a final water content of ~13–25% is reached (9, 11). The ripening process can take between 1 and 11 days to complete depending on factors such as colony size, climatic conditions, and botanical origin of the nectar (12). Once the honey is ripe, it is capped off with a thin wax layer for long-term storage to protect the hive from nectar shortages when foraging is not possible due to unfavorable seasonal and climatic conditions. In the absence of forage, honey bee colonies would collapse within a few days without an adequate store of honey (13).

### Adaptive consumption based on health needs

Beyond providing food for the colony to maintain basic physiological functioning, the production of honey has additional nutritional benefits. The properties, both beneficial and detrimental, of foraged plant-derived compounds can vary significantly depending on the floral and geographic source. While an individual honey bee may forage from only one or a few floral sources, at the level of the colony and over the course of a season, a wide range of nectars can be collected, processed, and stored as honey, providing a more suitable and consistent diet across the colony (14). Additionally, honey bees have long been known to exhibit preferences for certain food sources over others including the specific position of one flower over another (15), and to adjust their choices amongst more and less profitable nectar sources based on the nutritional needs of the colony (16, 17). Foragers will also avoid nectar containing particular bacterial communities (18), and in laboratory studies will selectively choose among various types of honey depending on their health status (19). The capacity to store various products in the hive, including honey, resins, propolis, pollen, bee bread, and beeswax, is thought to allow honey bees to adapt their food source and behavior based on the health status of individuals or the colony in order to manage nutritional and health challenges (14).

### Detoxification and enhanced toxin tolerance

While foraged pollen and nectar offer a diverse array of beneficial chemicals, they can also contain considerable amounts of toxic phytochemicals such as some alkaloids, coumarins, flavanols, and saponins (20). Honey bees can also be exposed to toxic pesticides though nectar, with some extremely persistent in the environment; for example neonicotinoid insecticides can be taken up by plants from environmental reservoirs in water and soil for years after their application (21). The processing of nectar into honey assists detoxification both directly and indirectly. Directly, the enzyme β-glucosidase is added by secretions from the honey bee hypopharyngeal gland and breaks down glycosidic toxins (9). Indirectly, the variety of floral resources collected by the colony allows them to be mixed and diluted thereby reducing the potential toxicity from any one nectar source (22). Ripening honey is also exposed to temperatures in the hive maintained around 35°C, which has been shown to decrease phenolic content including harmful phenolics present in some toxic nectars (23). Maintaining honey at this temperature is thought to similarly deactivate other compounds including potential toxins, reducing the concentration that must then be cleared via further enzymatic processing by the honey bee (22) but likely also reducing potentially beneficial compounds.

Honey consumption has been demonstrated to enhance honey bee tolerance to ingested natural and synthetic toxins. Specific components of honey that have been identified to play a role in the detoxification process include the flavonoid quercetin, the phenolic acid *p-*coumaric acid, and the plant hormone abscisic acid. RNA-sequencing analysis revealed that both low and high levels of quercetin in larval diets induced upregulation of multiple cytochrome P450 monooxygenases, one of the major enzyme subfamilies responsible for the metabolism of toxins in honey bees (24). Diet supplementation with *p-*coumaric acid increased metabolism of the acaricide coumaphos (25), and increased the survival of honey bees exposed to the insecticide tau-fluvalinate (26). Supplementation with abscisic acid increased the tolerance of honey bees to the pesticide carvacrol, and the acaricide oxalic acid (27). Various other phenolic acids and flavonoids present in honey including flavones, flavanones, and flavanols have also been shown to induce detoxification-related genes (25).

### Antioxidant-based enhancement of honey bee longevity

The accumulation of oxidative damage to lipids, proteins, and nucleic acids caused by reactive oxygen species (ROS) has been linked to aging and death in various organisms including the honey bee. Honey bee senescence accelerates with the transition from in-hive tasks to foraging behavior (28), presumed to be due to an increased metabolic rate to meet the requirements of hovering flight resulting in elevated production of several ROS including superoxide anions and hydroxyl radicals (29). Components of honey that exhibit antioxidant activity are thought to enhance the longevity of the honey bee by neutralizing ROS. The enzyme catalase, originating from pollen and nectar, metabolizes hydrogen peroxide to produce water and molecular oxygen. This protects honey bees from oxidative damage caused by high levels of hydrogen peroxide and works in combination with other antioxidant enzymes expressed in the honey bee gut such as superoxide dismutase and peroxidases (30). Phenolic compounds, peptides, vitamins, organic acids, and trace elements have also been identified as contributing to the antioxidant capacity of honey (31), which can vary significantly with floral source (32).

### Other diverse ecological benefits

Honey is thought to play a role in various other ecological functions including developmental regulation, immune system enhancement, coordination of the stress response, and supporting the honey bee gut microbiome. As diet plays a large role in honey bee caste determination, phytochemical components of honey have the potential to influence developmental pathways. While queen-destined larvae are fed only royal jelly, worker-destined larvae consume a diet that includes honey along with royal jelly and beebread (33). Although present in greater quantities in pollen, components of honey including *p*-coumaric acid and plant microRNAs have been shown to reduce ovary development and promote differentiation into workers (33, 34). The honey bee humoral immune system involves the production and secretion of antimicrobial peptides that are active against various pathogens including bacteria, fungi, viruses, and parasites (35). Diverse phytochemicals found in honey upregulate various antimicrobial peptides including hypenoptaecin (36), and apidaecin (33). Consumption of abscisic acid, a phytohormone commonly present in honey, has been implicated in coordinating honey bee stress response including stimulating innate immune defenses such as wound healing and phagocytosis activation (11), and inducing cold tolerance during overwintering (27). The community of microbes that live in the honey bee gut, referred to as the gut microbiome, play a role in various aspects of host health including contributing to digestion and nutrient intake, and out-competing harmful microbes (37). It is likely that consumption of honey supports the health of the honey bee gut microbiome, and studies investigating honey in the context of human gut health have shown that honey oligosaccharides can promote the growth of beneficial lactobacilli and bifidobacteria (38), which are also members of the honey bee gut (39).

## The mechanisms and ecological significance of antimicrobial activity

### Osmotic pressure, low water activity, and hydrogen peroxide

Honey is a supersaturated sugar solution, rendering it unfavorable for microbial growth via two mechanisms. The first is a low water content that results in high osmotic pressure, drawing water out of microorganisms and inhibiting their growth. The exact water content of honey can be influenced by various factors including the floral origin of the nectar and seasonal and climatic conditions, but normally ranges from between 13 and 25%, with around 17% being optimal as above this honey is vulnerable to fermentation by osmophilic yeasts (9). The second mechanism is low water activity resulting from the high concentration of sugar, which makes water unavailable for microorganisms to utilize for growth. The water activity of honey typically ranges from 0.5 to 0.65, which is lower than the amount needed for most bacteria (0.90), yeasts (0.80), and molds (0.70) to grow (9, 37). A water activity below 0.61 will also inhibit the growth of osmophilic yeasts (38).

Immature honey that has not yet reached a sufficient sugar concentration, and honey that has been diluted after processing, however, would still be vulnerable to microbial degradation without additional antimicrobial mechanisms. Nurse bees generally dilute honey with water before feeding it to larvae and adults due to its high viscosity (11) and ensuring protection of this diluted honey is essential. Glucose oxidase (GOX) is an enzyme that is added to nectar from the hypopharyngeal glands of honey bees during processing (39) and converts glucose to gluconolactone in diluted honey, in turn yielding gluconic acid and hydrogen peroxide (40). GOX is highly expressed in worker bees across developmental stages reaching its highest level in nectar processors and foragers (41). Gluconic acid discourages microbial overgrowth by lowering the pH of honey. Over thirty other organic acids have been identified in honey including acetic, citric, formic, lactic, and malic, which contribute to keeping the pH between 3.2 and 4.5, however gluconic acid is predominant representing 70–90% of all honey acids (9, 42, 43). The hydrogen peroxide produced by GOX activity destroys microbes via free hydroxyl radical production, which causes oxidation of microbial lipids, proteins, and nucleic acids (44). Maximum production of hydrogen peroxide is reached at 30–50% dilution, depending on the variety of honey (45, 46).

### Plant-, honey bee-, and microbiota-derived compounds

As an incredibly complex mix comprised of up to 200 different substances (47), it is no surprise that components of honey responsible for antimicrobial effects are increasingly being identified. These components originate from foraged plant sources, endogenous honey bee secretions, and associated microbiota, and are often specific to particular varieties of honey. One of the most well-known plant derived components is methylglyoxal (MGO), identified as the main antibacterial component of manuka honey produced from the nectar of certain *Leptospermum* species native to New Zealand and Australia (48). MGO is thought to crosslink proteins and DNA, damage cell membranes, and alter the structure of bacterial fimbriae and flagella, limiting their adherence and motility (49). Among other plant-derived components associated with antimicrobial activity are secondary metabolites such as polyphenols, flavonoids, volatile compounds, and alkaloids present in nectar (9). These diverse phytochemicals have evolved as innate plant protection systems against stresses including microbial infection and degradation (50) and thus likely perform similar ecological functions in honey. Many of these components are found at concentrations that are not sufficient to account for significant antimicrobial activity however, and are thought to work synergistically with other honey components including hydrogen peroxide (51).

Among honey bee-derived antimicrobial components are the antimicrobial peptide bee defensin-1, and major royal jelly protein-1. Bee defensin-1, also known as royalisin, is found in honey bee haemolymph as well as the hypopharyngeal gland, from where it is introduced to honey during processing (51). It is active against both gram-positive and gram-negative bacteria and is also implicated in the ability of honey to damage biofilms (52, 53). Although the mechanism of action has not yet been confirmed, bee defensin-1 is thought to act in a similar way to defensins from other species by disrupting the bacterial cell membrane, resulting in the inhibition of DNA, RNA, and protein synthesis (47). Major royal jelly protein-1 (MRJP1) is the most abundant protein found in both royal jelly and honey, and it is also introduced from the hypopharyngeal gland during nectar processing (54). Upon cleavage, MRJP1 yields the antimicrobial peptides jellein-1, jellien-2, and jellein-4, which have been shown to cause cell wall lysis and death in bacteria (55, 56). Finally, recent work suggests that honey can be a reservoir of microbe-produced antimicrobial compounds including antimicrobial peptides, surfactants, and proteolytic and cell wall-degrading enzymes that are produced by antagonistic microbial interactions in plant nectars, the honey bee gut, and the honey itself (57). The compounds produced by these microbes may account for the target-specific antimicrobial effects observed with honey that cannot be due to the non-specific broad-spectrum action caused by the principal antimicrobial mechanisms of honey. Antimicrobial peptides produced by the bacteria *Lactobacillus kunkeei* (58) and *Bacillus subtilis* (59) isolated from honey samples have shown activity against human, hive, and food spoilage pathogens including bacteria and fungi.

### Protection against significant hive pathogens

While the antimicrobial activity of honey against human pathogens has been extremely well-documented, there are far fewer studies that have assayed honey against ecologically relevant hive pathogens. American Foulbrood (AFB) is a highly contagious disease capable of wiping out the entire hive, and is acquired when larvae ingest spores of the bacterium *Paenibacillus larvae* that then germinate in the gut, causing infected larvae to die in the brood cell (60). The endospores produced by *P. larvae* are highly resilient and capable of surviving for many years, and infected hives and equipment often need to be burned to ensure that they are destroyed. Monofloral black locust, canola, citrus, clover, cotton, heather, honeydew, linden, and sunflower honey and polyfloral Romanian honey have been shown to inhibit the growth of *P. larvae* vegetative cells at varying concentrations (61–64) (Table 2). The antimicrobial peptide bee defensin-1 has also been identified as a specific component of honey with activity against *P. larvae* (86). European Foulbrood (EFB) is another common bacterial disease that is acquired when larvae ingest food contaminated with the bacterium *Melissococcus plutonius* which begins reproducing in the midgut, deriving nutrients from the larvae and causing starvation (60). Secondary invading bacteria are often present in weakened colonies suffering from EFB including *Bacillus pumilus, Brevibacillus laterosporus, Enterococcus faecalis*, and *Paenibacillus alvei* (67). Antimicrobial activity against EFB and/or its secondary invaders has been reported for monofloral almond, eucalyptus, manuka, and orange honey and polyfloral Argentinean, Australian, Brazilian, Cuban, Romanian, Spanish, and Turkish honey (63, 68–73, 87, 88) (Table 2).

**Table 2:** Honey varieties with antimicrobial activity against hive pathogens.

**Disease**	**Pathogen**	**Honey varieties**	**Effect on pathogen**	**References**
**Bacteria**				
American foulbrood	*Paenibacillus larvae*	Black Locust (*Robinia pseudoacacia*)	Growth inhibition	(65–68)
		Canola/Rape (*Brassica rapa*)		
		Citrus (*Citrus spp*.)		
		Clover (*Trifolium alexandrium*)		
		Cotton (*Gossypin barbadens*)		
		Heather (*Calluna vulgaris*)		
		Honeydew		
		Linden (*Tillia sp*.)		
		Romanian Polyfloral		
		Sunflower (*Helianthus annuus*)		
European foulbrood	*Melissococcus plutonius*	Black Locust (*Robinia pseudoacacia*)	Growth inhibition	(67)
		Romanian Polyfloral		
		Sunflower (*Helianthus annuus*)		
European foulbrood (Secondary invaders)	*Bacillus pumilus*	Black Locust (*Robinia pseudoacacia*)	Growth inhibition	(67)
		Romanian Polyfloral		
		Sunflower (*Helianthus annuus*)		
	*Brevibacillus laterosporus*	Black Locust (*Robinia pseudoacacia*)	Growth inhibition	(67)
		Romanian Polyfloral		
		Sunflower (*Helianthus annuus*)		
	*Enterococcus faecalis*	Activon (*Leptospermum spp*.)	Growth inhibition	(69–76)
		African Beech (*Faurea saligna*)		
		Algarrobo (*Prosopis nigra*)		
		Almond (*Prunus dulcis*)		
		Anzer Brand Turkish Honey		
		Argentinian Polyfloral		
		Australian Polyfloral		
		Banksia (*Banksia spp*.)		
		Blackbutt (*Eucalyptus patens*)		
		Bottlebrush (*Callistemon spp*.)		
		Brazilian Polyfloral		
		“Capilano” Brand Australian Honey		
		Citrus (*Citrus limon*)		
		Cuban Polyfloral		
		Eucalyptus (*Eucalyptus spp*.)		
		Jarrah (*Eucalyptus marginata*)		
		Lemon (*Citrus limon*)		
		Manuka (*Leptospermum scoparium*)		
		Manuka (*Leptospermum subtenue*)		
		Marri (*Corymbia calophylla*)		
		Medlar (*Mespilus germanica*)		
		Mexican polyfloral		
		Moort (*Eucalyptus platypus*)		
		Orange (*Citrus spp*.)		
		Prickly Pear (*Optunia spp*.)		
		Rhododendron (*Rhododendron spp*.)		
		Rubus (*Castnea sativa*)		
		Spanish polyfloral		
		Turkish polyfloral		
		Wandoo (*Eucalyptus wandoo*)		
	*Paenibacillus alvei*	Black locust (*Robinia pseudoacacia*)	Growth inhibition	(67)
		Romanian Polyfloral		
		Sunflower (*Helianthus annuus*)		
**Fungi**				
Nosemosis	*Nosema apis*	Manuka (*Leptospermum scoparium*)	Decreased spore viability	(77)
		New Zealand Polyfloral		
	*Nosema ceranae*	Black Locust (*Robinia pseudoacacia*)	Decreased spore load in infected bees	(19)
		Sunflower (*Helianthus annuus*)		
Stonebrood	*Aspergillus flavus*	Almond (*Prunus dulcis*)	Growth inhibition	(72, 78, 79)
		Lemon (*Citrus limon*)		
		Manuka (*Leptospermum scoparium*)		
		Medlar (*Mespilus germanica*)		
		Orange (*Citrus spp*.)		
		Prickly Pear (*Optunia spp*.)		
		Unspecified Pakistani Honey		
		Unspecified Nigerian Honey	Growth and sporulation reduction	(80)
		Orange (*Citrus spp*.)	Prevention of aflatoxin production	(81)
	*Aspergillus fumigatus*	Unspecified Nigerian Honey	Growth and sporulation reduction	(80)
		Beninese Polyfloral Honey	Growth inhibition	(78, 79, 82)
		Manuka (*Leptospermum scoparium*)		
		Unspecified Pakistani Honey		
	*Aspergillus niger*	Acacia (*Robinia pseudoacacia*)	Growth inhibition	(78, 83–85)
		“Apis” Brand Himalayan Honey		
		“Dabur” Brand Indian Honey		
		“Khadi” Brand Indian Honey		
		Malaysian Tualang Polyfloral		
		Siddar (*Ziziphus jujube*)		
		Unspecified Pakistani Honey		
	*Aspergillus parasiticus*	Orange (*Citrus spp*.)	Prevention of aflatoxin production	(81)
		Beninese Polyfloral Honey	Growth inhibition	(82)

Nosemosis is the most common disease of adult honey bees and is acquired when spores of the microsporidian fungi *Nosema ceranae or Nosema apis* are ingested. The spores then germinate in the midgut causing difficulty with digestion, increasing susceptibility to viral infection and weakening the honey bees, leading to reductions in lifespan, colony health, and colony performance (60, 74). A study testing the viability of *N. apis* spores found that it significantly decreased after only 3 days in the presence of New Zealand polyfloral honey compared to 1 month in manuka monofloral honey and 21 days in sugar syrup (75), while another study found infected honey bees ingesting sunflower honey had significantly decreased spore loads of *N. ceranae* compared to honey bees ingesting honeydew honey (19) (Table 2). Stonebrood is a less common fungal infection acquired when larvae ingest spores of *Aspergillus* species. Stonebrood can be fatal when mycotoxins produced by the fungi kill and mummify the larvae before they hatch (60). While no studies have determined whether honey is capable of killing *Aspergillus* spores, monofloral acacia, almond, lemon, manuka, medlar, orange, prickly pear, and siddar honey and polyfloral Beninese and Malaysian honey have been shown to inhibit the growth of various *Aspergillus* species including *A. flavus, A. fumigatus, A. niger*, and *A. parasiticus* (69, 76–79, 83, 89) (Table 2). Nigerian honey of unspecified botanical origin has been observed to reduce growth and sporulation in *A. flavus* and *A. fumigatus* (84), and orange honey has been observed to prevent aflatoxin production by *A. flavus* and *A. parasiticus* (85). This demonstrates the impressive ability of honey to protect the hive even from a ubiquitous environmental fungus that is capable of growth at low water activity and elevated temperatures.

## Summary and conclusions

Honey plays a fundamental role in maintaining colony health. Beyond simple nutrition, honey consumption has far-ranging impacts on toxin tolerance, honey bee longevity, developmental regulation, immunity, stress response, and resilience to pathogens. Beekeeping practices that substitute sugar solutions for honey may provide the hive with general nutrition, but this could have consequences on numerous aspects of honey bee and hive health. There is still a great deal we do not fully understand about how and why honey bees create and utilize honey. This includes by what mechanisms honey bees alter their foraging behaviors based on colony needs, how the microbiota of foraged plant components and the honey bee gut contribute to the functional properties of honey, whether and how various phytochemicals, endogenous secretions, and other components of honey work synergistically to exert their full effects, and how diverse foraging sources impact bee and hive health. Answering these questions could go a long way toward ensuring that honey bees and beekeepers alike prosper for decades to come.

## Author contributions

This article was written by KF. Reviewed and edited by EF, ER, KS, NC, and DC. All authors contributed to the article and approved the submitted version.

## Funding

Honey research projects undertaken by our team were supported by the NSW Bushfire Industry Recovery Package Sector Development Grant (BIP-SDG-135).

## Conflict of interest

The authors declare that the research was conducted in the absence of any commercial or financial relationships that could be construed as a potential conflict of interest.

## Publisher's note

All claims expressed in this article are solely those of the authors and do not necessarily represent those of their affiliated organizations, or those of the publisher, the editors and the reviewers. Any product that may be evaluated in this article, or claim that may be made by its manufacturer, is not guaranteed or endorsed by the publisher.
